# Emerging high-risk ST101 and ST307 carbapenem-resistant *Klebsiella pneumoniae* clones from bloodstream infections in Southern Italy

**DOI:** 10.1186/s12941-020-00366-y

**Published:** 2020-06-01

**Authors:** Daniela Loconsole, Marisa Accogli, Anna Lisa De Robertis, Loredana Capozzi, Angelica Bianco, Anna Morea, Rosanna Mallamaci, Michele Quarto, Antonio Parisi, Maria Chironna

**Affiliations:** 1 Department of Biomedical Sciences and Human Oncology, Hygiene Unit, University of Bari “Aldo Moro”, Bari, Italy; 2 Istituto Zooprofilattico Sperimentale della Puglia e della Basilicata, Foggia, Italy; 3 Department of Biosciences, Biotechnology and Biopharmaceutics, University of Bari “Aldo Moro”, Bari, Italy

**Keywords:** Carbapenem-resistant *Klebsiella pneumoniae*, Bloodstream infections, Drug resistance, ST101, ST307, Whole genome sequencing, Resistome, Virulome

## Abstract

**Background:**

Carbapenem-resistant *Klebsiella pneumoniae* (CR-KP) is an urgent public health issue in Italy. This pattern of resistance is due mainly to dissemination of carbapenemase genes. Molecular characterization of carbapenem-resistant *Klebsiella pneumoniae* (CR-KP) strains was performed over a three-year period. In-depth analysis was performed on a subset of emerging CR-KP ST101 and ST307 clones.

**Methods:**

A prospective study was performed on 691 patients with CR-KP bloodstream infections hospitalized in 19 hospitals located in three large provinces in Southern Italy. Carbapenemase genes were identified via genotyping methods. Multi-locus sequence typing (MLST) and Whole Genome Sequencing (WGS) were carried out on ST101 and ST307 isolates.

**Results:**

Among the CR-KP isolates, *bla*_KPC_ was found in 95.6%, *bla*_VIM_ was found in 3.5%, *bla*_NDM_ was found in 0.1% and *bla*_OXA-48_ was found in 0.1%. The *bla*_KPC-3_ variant was identified in all 104 characterized KPC-KP isolates. MLST of 231 representative isolates revealed ST512 in 45.5%, ST101 in 20.3% and ST307 in 18.2% of the isolates. cgMLST of ST307 and ST101 isolates revealed presence of more than one beta-lactam resistance gene. Amino acid substitution in the chromosomal colistin-resistance gene *pmrB* was found in two ST101 isolates.

**Conclusions:**

ST512 is widespread in Southern Italy, but ST101 and ST307 are emerging since they were found in a significant proportion of cases. Aggressive infection control measures and a continuous monitoring of these high-risk clones are necessary to avoid rapid spread of CR-KP, especially in hospital settings.

## Background

The spread of antimicrobial resistance (AMR) was recently identified by the World Health Organization (WHO) as a priority threat to human health. In particular, carbapenem-resistant Enterobacteriaceae (CRE) were included in the most critical group of antibiotic-resistant Gram-negative bacteria [1], as infections with these organisms show high mortality rates, especially in cases of bloodstream infections (BSIs) [2, 3]. CRE infections, mainly with carbapenem-resistant *Klebsiella pneumoniae* (CR-KP), have become endemic in several countries [4]. In Italy, an abrupt and notable increase in the proportion of CR-KP invasive infections was reported in 2010 (from 1.2% in 2009 to 15.2% in 2010), making CR-KP the main antibiotic-resistance threat [5–8]. A peak of prevalence was reached in 2016 (33.9%), but a significant decreasing trend was reported in 2017 (29.7%) [4].

Nationwide surveillance in the period 2014–2017 showed that 95.2% of the BSIs caused by carbapenemase-producing Enterobacteriaceae (CPE) were due to KP strains carrying the *Klebsiella pneumoniae* carbapenemase (KPC) enzyme [9]. Metallo-beta-lactamases, including NDM (New Delhi metallo-beta-lactamase) and VIM (Verona integron-encoded metallo-beta-lactamase) enzymes, were identified in 1.9% of the BSIs, whereas OXA-48 (carbapenem-hydrolysing oxacillinase-48) was found in 1.2% of the cases. KP strains coproducing several classes of carbapenemases were identified in 1.3% of the BSIs. Among the KPC-KP isolates in Italy, the *bla*_KPC-3_ gene was the most prevalent [10–16].

In our country, recent studies on the molecular epidemiology of KPC-KP from invasive infections showed an expansion of strains belonging to sequence types (STs) 512 and 258 of the hyperepidemic clonal complex (CC) 258 [7, 17], which was detected for the first time in Italy in 2008 [18].

Recent epidemiological reports showed that ST101 and ST307 are emerging in several countries; therefore, these STs are considered candidates for becoming the prevalent high-risk CR-KP in the near future [10, 16, 17, 19–25]. Genetic features of these clones may contribute to their adaptation to hospital environments and to the human host, both of which result promote further spread of antibiotic resistance [26, 27].

In Italy, the emerging ST101 and ST307 have been rarely reported in patients with invasive infections to date [7, 10, 16, 17, 28]. Few data are available on the molecular characteristics of the CR-KP strains involved in BSIs in the Puglia region in Southern Italy. Recently, Calia et al. reported that 68 out of 75 strains (90.7%) collected from patients with sepsis in Puglia and Basilicata harboured the *bla*_KPC-3_ gene and that ST512 was the main sequence type detected in the CR-KP isolates from several clinical samples [28]. ST307 was reported in five cases, whereas none of the characterized strains belonged to ST101.

In this study, we present the results of molecular characterization of CR-KP strains isolated from hospitalized patients with BSIs detected during the 3-year period from 2014 to 2016 in three large provinces of the Puglia region in Southern Italy. The observed increasing prevalence of ST101 and ST307 in the study period prompted us to conduct in-depth analyses of the lineage relatedness, resistance determinants, and core and accessory genome contents of these clones via whole-genome sequencing.

## Methods

### Study design

A prospective study was designed at the University Hospital Policlinico in Bari, Italy, after that the Italian Ministry of Health (MoH) issued a directive to monitor and control CPE in 2013 (MoH DGPRE n°4968 26/02/2013). The study involved three provinces in the Puglia region (Bari, Brindisi and Barletta-Andria-Trani), which have a population of about 2 million inhabitants, representing half of the overall regional population (source demographic data: ISTAT, 2018; http://demo.istat.it/pop2018/index.html). Nineteen hospitals were enrolled: eight were located in the province of Bari, six in Brindisi, and five in Barletta-Andria-Trani. During the 2014–2016 period, the microbiology laboratories of the hospitals involved in the study collected the consecutive non-replicate clinical CR-KP isolates from the blood of patients with BSIs hospitalized in intensive care units (ICUs) or medical or surgical wards. The collected strains included those with intermediate resistance or resistance to imipenem and/or meropenem based on the EUCAST breakpoints (http://www.eucast.org/clinicalbreakpoints/). All of these isolates were then sent to the Laboratory of Molecular Epidemiology and Public Health of the Hygiene Unit of the University Hospital Policlinico of Bari for genotypic confirmation of their carbapenem resistance and identification of their carbapenemase genes. Further molecular characterization was performed on a representative subset of the isolates. For each hospitalized patient, demographic data (e.g., sex, age, etc.), the name and province of the hospital, the type of ward, the presumptive source of the BSI, and phenotypic resistance data were collected.

### Molecular characterization of the isolates

All of the CR-KP isolates were tested using a commercial multiplex real-time PCR kit (GeneXpert platform, Cepheid, Sunnyvale, CA, USA) to identify the following carbapenemase-encoding genes: KPC (*bla*_KPC_), VIM (*bla*_VIM_), NDM (*bla*_NDM_), IMP (*bla*_IMP_) and OXA-48 (*bla*_OXA-48_). Variant identification was performed on 104 representative KPC-KP isolates from all the hospitals involved and the 3-years period of the study via PCR, as previously described [7], and subsequent sequencing. Allelic variants were identified via the BLAST program on the National Center for Biotechnology Information website (http://blast.ncbi.nlm.nih.gov).

Multi-locus sequence typing (MLST) of 231 representative CR-KP isolates was performed by amplifying and sequencing internal fragments derived from seven specific housekeeping KP genes (*rpoB*, *gapA*, *mdh*, *pgi*, *phoE*, *infB*, and *tonB*) [29]. The allelic profiles were compared with those included in the electronic database of the Pasteur Institute to identify the ST (bigsdb.web.pasteur.fr/klebsiella/klebsiella.html).

### Whole Genome Sequencing (WGS) of the ST307 and ST101 isolates

Seven CR-KP isolates, previously typed as ST101 (n = 2) and ST307 (n = 5) were available for WGS, which was performed at the Istituto Zooprofilattico di Puglia e Basilicata, Foggia (Italy) using an Illumina MiSeq (Illumina Inc., San Diego, CA, USA) with a paired-end run of 2 by 250 bp after Nextera XT paired-end library preparation. Sequencing reads were assembled using SPAdes genome (version 3.12).

The virulence- and AMR-associated genes located on both the bacterial chromosome and on mobile genetic elements in the characterized CR-KP isolates were identified by uploading the assembled contigs into the online tool BIGSdb-Kp (http://bigsdb.pasteur.fr/klebsiella/klebsiella.html). The plasmid incompatibility types were identified via PlasmidFinder [30]. A 95% identity cut-off was used.

For each isolate, a panel of 634 genes, defined as the strict cgMLST set, was identified submitting the draft genomes to the BIGSdb-Kp (http://bigsdb.pasteur.fr/klebsiella/klebsiella.html) [31].

## Results

During the 3-year study period, a total of 691 patients with BSIs were included in the study. Of these, 40.2% (n = 278) were in the ICUs, 39.5% (n = 273) were in the medical wards and 13.6% (n = 94) were in surgical wards. The remaining 6.7% (n = 46) of the BSIs occurred in patients whose ward was not known. The mean age was 63.8 years (range: 0–97 years), and 444 patients (64.3%) were men. Among the included cases, the origins of the BSIs were distributed as follows: central/peripheral venous catheter infections, 12.9% (n = 89); ventilator-associated pneumonia (VAP), 11.3% (n = 78); urinary tract infections, 8.2% (n = 57); pneumonia, 6.8% (n = 47); skin and tissue infections, 3.9% (n = 27); abdominal infections, 2.8% (n = 19); surgical site infections, 2.5% (n = 17). In 26.9% (n = 186) of the cases, the bacteraemia was of unknown origin, while data regarding the infection site were missing for 24.7% (n = 171) of the cases.

All the 691 collected isolates were subjected to detection of carbapenemase genes (Table 1). Over these years, the following resistance genes were detected in CR-KP isolates at the given frequencies: *bla*_KPC_, 95.6% (n = 661); *bla*_VIM_, 3.5% (n = 24); *bla*_NDM_, 0.1% (n = 1); *bla*_OXA-48_, 0.1% (n = 1). The *bla*_IMP_ gene was not detected. Carriage of multiple carbapenemase genes was reported in four isolates (3 *bla*_KPC_ +* bla*_VIM_ and 1 *bla*_NDM_ +* bla*_OXA-48_). Of the three *bla*_KPC_ +* bla*_VIM_ strains, two were isolated from male patients of 74 and 78 years, who were hospitalized in the ICU of the same hospital (province of Bari) in June and July 2015. In both cases, the origin of infection was VAP. The third *bla*_KPC_ +* bla*_VIM_ strain was isolated in an 80-year-old female hospitalized in 2016 in a medical ward of another hospital in the province of Bari, and her bacteraemia originated from a urinary tract infection. The *bla*_NDM_ +* bla*_OXA-48_ isolate was identified in a 70-year-old male hospitalized in an ICU in 2016 in the province of Brindisi, and his bacteraemia was of unknown origin.

**Table 1:** Carbapenemase genes detected in 691 carbapenem-resistant *K. pneumoniae* isolates from bloodstream infections, sorted by isolation year, ward and origin of bacteremia, Puglia, Southern Italy

Isolation year	N. of isolates	Carbapenemase gene	Ward	Origin of bacteremia^a^
2014	150	KPC (n = 145) VIM (n = 5)	ICU (n = 60) Medical ward (n = 58) Surgical ward (n = 17) Unknown (n = 10) ICU (n = 2) Medical ward (n = 2) Surgical ward (n = 1)	Bacteremia of unknown origin (n = 33) Central/peripheral venous catheter infection (n = 28) Ventilator-associated pneumonia (n = 13) Skin and tissues infection (n = 12) Urinary tract infection (n = 10) Pneumonia (n = 7) Abdominal infection (n = 5) Surgical site infection (n = 3) Urinary tract infection (n = 1) Bacteremia of unknown origin (n = 1) Central/peripheral venous catheter infection (n = 1)
2015	179	KPC (n = 165) KPC + VIM (n = 2) VIM (n = 11) NDM (n = 1)	ICU (n = 57) Medical ward (n = 58) Surgical ward (n = 23) Unknown (n = 27) ICU (n = 2) ICU (n = 2) Medical ward (n = 5) Surgical ward (n = 4) Medical ward (n = 1)	Bacteremia of unknown origin (n = 39) Central/peripheral venous catheter infection (n = 24) Ventilator-associated pneumonia (n = 20) Pneumonia (n = 16) Urinary tract infection (n = 16) Skin and tissues infection (n = 4) Abdominal infection (n = 2) Surgical site infection (n = 2) Ventilator-associated pneumonia (n = 2) Ventilator-associated pneumonia (n = 3) Urinary tract infection (n = 3) Bacteremia of unknown origin (n = 2) Pneumonia (n = 2) Skin and tissues infection (n = 1)
2016	362	KPC (n = 351) VIM (n = 8) KPC + VIM (n = 1) NDM + OXA-48 (n = 1) OXA-48 (n = 1)	ICU (n = 149) Medical ward (n = 144) Surgical ward (n = 49) Unknown (n = 9) ICU (n = 4) Medical ward (n = 4) Medical ward (n = 1) ICU (n = 1) ICU (n = 1)	Bacteremia of unknown origin (n = 105) Central/peripheral venous catheter infection (n = 34) Abdominal infection (n = 11) Urinary tract infection (n = 26) Surgical site infection (n = 12) Pneumonia (n = 22) Ventilator-associated pneumonia (n = 40) Skin and tissues infection (n = 10) Bacteremia of unknown origin (n = 5) Central/peripheral venous catheter infection (n = 2) Urinary tract infection (n = 1) Bacteremia of unknown origin (n = 1) Abdominal infection (n = 1)

^a^Data regarding the infection site were missing for 171 cases

The *bla*_KPC_ variant was identified in 104 KPC-KP isolates. In particular, 19/145 (13%) isolates collected in 2014, 27/165 (16%) in 2015 and 58/351 (16%) in 2016 were subjected to variant detection. All of the strains carried the *bla*_KPC-3_ variant. MLST was performed on 231 isolates (Table 2). In the 50.7% of the characterized isolates, ST512 and ST258, which belong to CC258, were identified. Of the isolates, 20.3% and 18.2% belonged to ST101 and ST307, respectively. Significant proportions of ST101 and ST307 strains were observed over the study period. Peaks for ST101 and ST307 were reported in 2016 (29.5% and 23.8%, respectively). Other STs were reported in 10.8% of the isolates.

**Table 2:** Distribution of sequence type of 231 characterized carbapenem-resistant *Klebsiella pneumoniae* by year of isolation, Puglia region, Southern Italy

Year	Number of CR-KP characterized/number of isolates (%)	Sequence type				
		512	258	101	307	Other
		N. (%)	N. (%)	N. (%)	N. (%)	N. (%)
2014	54/150 (36.0)	30 (55.5)	3 (5.5)	7 (12.9)	6 (11.2)	8 (14.9)
2015	72/179 (40.2)	45 (62.5)	1 (1.4)	9 (12.5)	11 (15.3)	6 (8.3)
2016	105/362 (29.0)	30 (28.6)	8 (7.6)	31 (29.5)	25 (23.8)	11 (10.5)
Total	231/691 (33.4)	105 (45.5)	12 (5.2)	47 (20.3)	42 (18.2)	25 (10.8)

**Fig. 1:**
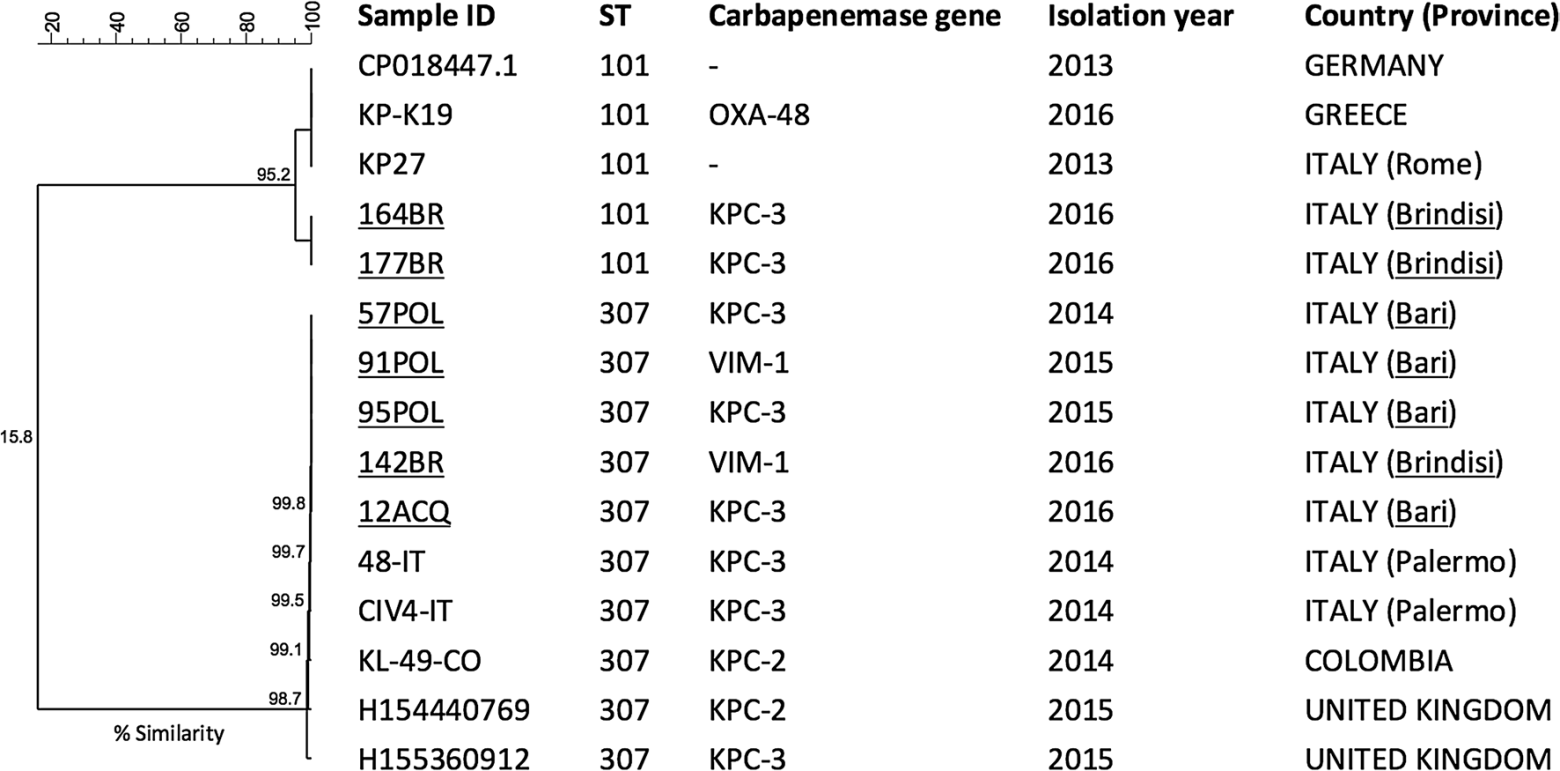
Phylogenetic tree of the carbapenem-resistant *K. pneumoniae* isolates characterized via cgMLST in Puglia region, Southern Italy. Isolates from Puglia are underlined. The sequence type (ST) is shown after the isolates name (Sample ID)

WGS was performed on five ST307 and two ST101 isolates. Based on EUCAST guideline, all of these strains were multidrug-resistant. A phylogenetic tree using cgMLST is shown in Fig. 1. The isolates of the present study were compared with other representative ST307 and ST101 present in the NCBI Pathogen detection database (https://www.ncbi.nlm.nih.gov/pathogens/). Very high similarity was observed among the ST307 strains, whereas, ST101 strains identified in Puglia region were phylogenetically more distant from the strains identified in Italy and in other countries. Among the isolates identified in Puglia region, all the ST101 carried the KPC-3 variant, whereas the five ST307 showed the VIM-1 variant in two cases and the KPC-3 variant in three cases. The classes of antimicrobial resistance genes and the plasmid replicons identified in the characterized isolates (i.e., a resistome analysis) are shown in Table 3. All of the characterized isolates carried multiple beta-lactam resistance genes. A plasmid replicon analysis revealed the presence of several plasmid replicon-types among the characterized isolates. The highest number of plasmid replicon-types was found in the ST101 isolates (n = 8; 6 Inc types and 2 Col types). The most frequently identified plasmid replicon-types were IncFII(K) (n = 6 isolates), IncFIB(K) (n = 4 isolates), IncR (n = 4 isolates) and the small ColRNAI plasmid (n = 4 isolates). In addition, plasmid types IncFIB, IncFII, IncQ1, and Col440II were exclusively detected in the ST101 isolates, while plasmid types IncFIB(K), IncFIB(Mar), IncFIB(pQil) and IncN were detected only in ST307 isolates.

**Table 3:** Resistome analysis of characterized carbapenem-resistant *K. pneumoniae* isolates from Puglia, Southern Italy

Antibiotic class	Gene^a^	Sequence type						
		ST307					ST101	
		57POL	91POL	95POL	142BR	12ACQ	164BR	177BR
Aminoglycoside	*aac(3)*-*IIa*-	c2	c2	c2	c2	c2		
	*aac(6′)*-*Ib*	b-cr	b6	b-cr	b-p	b-cr		
	*aph(3′)*-*Ia*			a1				
	*aph(3′′)*-*Ib*	2		2		2	2	2
	*aph(6)*-*Id*	1		1		1	1	1
β-lactams	*bla*_CTX-M-_	15	15	15		15		
	*bla*_SHV-_	28	55	28		28	1	1
	*bla*_OXA-_	1	1,9	1	9			
	*bla*_VIM-_		1		1			
	*bla*_TEM-_		1	1	1	1	1	1
	*bla*_KPC-_	3		3		3	3	3
Fluoroquinolones	*gyrA*	9	9	9	9	9	1	
	*gyrB*	1	1	1	1	1		1
	*parC*	25	25	25	25	25	25	25
	*parE*	1	1	1	1	1	1	1
	*qnr*	B1	B1	B1		B1	S1	
Efflux pumps	*acrA*	114	114	114	114	114	23	12
	*acrB*	41	41	41	41	41	27	
	*acrR*	1		1		1	2	
	*marA*	1	1	1	1	1	1	5
	*marR*	1	1	1	1	1	10	2
	*soxS*	1	1	1	1	1	2	6
	*soxR*	1	1	1	1	1	2	1
	*ramA*	2	2		2	2	6	7
	*ramR*						14	
	*rob*	3	3	3	3	3	3	1
	*sdiA*	2	2	2	2	2	1	2
	*fis*	1	1	1	1	1	1	1
	*envR*	20	20	20	20	20	3	2
	*oqxA*	136	136	136	136	136	24	12
	*oqxB*	156	156	156	156	156	26	12
	*oqxR*	4	4	4	4	4	15	4
	*rarA*	20	20	20	20	20	14	8
Plasmid replicon types^b^	IncFIA(HI1)		Χ		Χ		Χ	Χ
	IncFIB						Χ	Χ
	IncFIB(K)	Χ	Χ	Χ		Χ		
	IncFIB(Mar)					Χ		
	IncFIB(pQil)	Χ				Χ		
	IncFII						Χ	Χ
	IncFII(K)	Χ	Χ	Χ		Χ	Χ	Χ
	IncN			Χ				
	IncQ1						Χ	Χ
	IncR		Χ		Χ		Χ	Χ
	ColRNAI		Χ		Χ		Χ	Χ
	Col440II						Χ	Χ

^a^The allele codes identified for each gene is shown in the Table

^b^For the plasmid replicon analysis, an X indicates the presence of a replicon type determined via PlasmidFinder (%ID threshold: 95% and a query vs. HSP length ratio of > 98%)

None of the isolates carried any plasmid-borne *mcr* genes that confer colistin resistance; however, amino acid substitution L213M in the colistin resistance-conferring chromosomal *pmrB* gene was identified in the two ST101 isolates (164BR and 177BR) (reference strain: CGA_000240185.2 in GenBank), which were also phenotypically resistant to colistin (minimal inhibitory concentrations (MICs) > 4 mg/L for both isolates).

Table 4 provides the virulence and heavy metal resistance genes identified in the characterized isolates. All but one isolate (142BR, belonging to ST307) harboured the *pco*ABCDERS and *sil*CERS heavy metal resistance genes. Isolate 142BR carried the *pbr*A heavy metal resistance gene, which was not present in any other isolates, while only isolate 12ACQ carried the *ter*CZ genes. Sequencing of the capsular loci showed that all the ST307 isolates carried the *wzi*173 allele, while the ST101 isolates carried the *wzi*149 alleles.

**Table 4:** Virulence and heavy metal resistance genes detected in carbapenem-resistant *K. pneumoniae* isolates from Puglia, Southern Italy

Virulence gene	Gene^a^	ST307					ST101	
		57POL	91POL	95POL	142BR	12ACQ	164BR	177BR
*fyu, irp, iuc, kfu*	*fyuA*	11	11	11	11	11	11	11
	*irp1*	37	37	37	37	37	37	37
	*iucA*						45	45
	*iucC*						33	33
	*kfuA*						3	3
	*kfuB*						7	7
	*kfuC*						6	6
*mrk*	*mrkA*	12		12		12	12	12
	*mrkA/1*	87		87		87	87	87
	*mrkB*	2		2		2	30	30
	*mrkC*	135		135		135	41	41
	*mrkC/1*	302		302		302	302	302
	*mrkD*	8		8		8	35	35
	*mrkF*	4		4		4	71	71
	*mrkH*	2		2		2		
	*mrkI*	4		4		4	28	28
	*mrkJ*	2		2		2	28	28
*ybt*	*ybtA*	38	38	38	38	38	38	38
	*ybtE*	5	5	5	5	5	5	5
	*ybtP*		25	25	25	25	25	25
	*ybtQ*	6	6	6	6	6	6	6
	*ybtS*	41	41	41	41	41	41	41
	*ybtT*	5	5	5	5	5	5	5
	*ybtU*	39	39	39	39	39	39	39
	*ybtX*	11	11	11	11	11	11	11
Heavy metal resistance gene	*pbrA*				2			
	*pcoA*	2	2	2		2	7	7
	*pcoB*	3	3	3		3	2	2
	*pcoC*	1	1	1		1	1	1
	*pcoD*	2	2	2		2	2	2
	*pcoE*	1	1	1		1	1	1
	*pcoR*	1	1	1		1	1	1
	*pcoS*	2	2	2		2	2	2
	*silC*	3	3	3		3	3	3
	*silE*	3	3	3		3	3	3
	*silR*	2	2	2		2	2	2
	*silS*	2	2	2		2	2	2
	*terC*					9		
	*terZ*					1		

^a^The allele codes identified for each gene is shown in the Table

## Discussion

During the study period, almost all of the CR-KP strains isolated from the patients with BSIs in the Puglia region (Southern Italy) carried the *bla*_KPC_ gene (96%), confirming that the production of the KPC-type carbapenemase was the most common carbapenem-resistance mechanism, as previously reported in Italy [7, 9] and Southern Italy [28]. In our study, the KPC-3 variant was found in all of the analysed KPC-KP isolates, and this is consistent with the finding that this variant is the most prevalent in other KP surveillance studies in Italy [7, 10, 16, 17, 28]. We found a higher proportion of *bla*_VIM_ and *bla*_NDM_ genes (3.6%) in the BSIs compared with the proportion reported by Iacchini et al. (1.9%) for Italian isolates [9]. Calia et al. identified the *bla*_VIM_ gene in 5.0% of CR-KP isolates. However, the data reported in the latter study, which was conducted in the Puglia and Basilicata regions (Southern Italy), included strains isolated from any infection site and not BSIs specifically [28]. Moreover, we found one isolate carrying the *bla*_NDM_ gene, whereas no NDM-KP was reported by Calia et al. [28]. Carriage of multiple carbapenemase genes is rarely reported [9], and our study followed this trend (0.6%). Molecular characterization of such isolates could be of particular interest. Unfortunately, the four isolates carrying multiple carbapenem-resistance genes included in our study were not characterized because they were not available for WGS.

Almost half of the CR-KP isolates included in the present study belonged to ST512. However, over the study period, we found an increasing proportion in BSIs with ST101 and ST307 strains, which accounted for approximately 20% and 18% of all the 231 characterized isolates, respectively. These STs have been rarely reported in Italy, except for in the report of Bonura et al. and in Fasciana et al. in which prevalence of 29% (27/94) and of 12% (3/25) were reported for ST307, respectively [10, 16]. Nevertheless, these data included CR-KP strains isolated from any site of infection or colonization and not BSIs specifically. Furthermore, Calia et al. found only five isolates belonging to ST307 (3.3%) [28], which is substantially lower than the prevalence of this ST in the present study. In Milan (Lombardy), ST307 was found in 4.4% of isolates [32], whereas no strains belonging to this ST were found in Sassari (Sardinia) [11]. These data regarding the spread of ST307 in Italy seem to suggest that this ST is replacing the predominant hyperendemic STs belonging to CC258 [33].

ST101 is an emerging nosocomial high-risk clone associated with infections with increased mortality compared with non-ST101 infections [26]. Moreover, ST101 is a high-risk for mediating further spread of carbapenem resistance [34, 35]. In Italy, this ST has generally been reported as sporadic [7, 10, 11, 16, 17, 32], with higher prevalence when the studied strains were isolated from any infection site [34, 36, 37]. We found that approximately 18% of the characterized strains belonged to ST101, while no isolates belonging to this ST were identified in the report of Calia et al. in Southern Italy [28]. All of the ST101 isolates identified in the present study were KPC-3-producing strains, whereas other Italian studies reported ST101 isolates that only produced the KPC-2 variant [7, 17, 34].

Our resistome analysis showed that all of the characterized isolates harboured genes conferring resistance to aminoglycosides, beta-lactams, and fluoroquinolones. In particular, the ST307 isolates carried a significantly higher number of resistance genes compared to the numbers in the ST101 isolates. However, no clear correlation between genetic background and phenotypic resistance in these strains could be established [32]. In the present study, three out of five ST307 isolates (57POL, 95POL, and 12ACQ) carried *bla*_CTX-M-15_, *bla*_SHV-28_ and *bla*_KPC-3_. This pattern has been previously reported in Italy [16, 27, 37]. Furthermore, the three isolates that carried *bla*_CTX-M-15_, *bla*_SHV-28_ and *bla*_KPC-3_ showed the same allelic profile for efflux pump genes, which differed from that identified in the ST101 isolates.

The analysis of virulence genes showed that the two ST101 isolates seemed to be potentially extremely virulent strains, as they carried the siderophore gene (*irp*1), the ferric uptake system (*kfu*ABC), the yersiniabactin siderophore cluster genes (*ybt*AEPQSTUX), and the mannose-resistant *Klebsiella*-like (type III) fimbriae cluster (*mrk*ABCDFHIJ). These virulence factors have been previously reported in only one KP-ST101 isolate identified in Northern Italy in 2013 [32]. All of the isolates characterized in our study harboured at least one heavy metal resistance gene, which, as previously suggested, can contribute to antibiotic-resistance gene dissemination and maintenance [38]. Non-antibiotic compound, such as metals, may contribute to the spread of antibiotic resistance through the co-selection process [39]. In fact, genes conferring resistance to heavy metals and antibiotics may coexist on the same genetic element (e.g. plasmid), which could promote a further dissemination of the antibiotic-resistance mechanisms [38]. Moreover, a single resistance mechanism, such as an efflux pump, could confer resistance to both antibiotics and metals (cross-resistance) [39].

Interestingly, we also identified many plasmids in the characterized isolates, especially IncF-type and ColRNAI plasmids. This finding is consistent with studies reporting that these plasmids are frequently associated with successful dissemination of carbapenemase, CTX-M-type extended-spectrum beta-lactamase, and plasmid-mediated quinolone resistance genes, as well as other virulence determinants in *Enterobacteriaceae* [40, 41].

When treating infections involving antimicrobial resistance (AMR), colistin is the last-resort antibiotic for treatment of patients with *Enterobacteriaceae* infections. Colistin resistance associated with plasmid-borne *mcr* genes in KP has been reported in Italy, although rarely [42]. Traditionally, colistin resistance in KP is associated with mutations in chromosomal genes [43]. Among the isolates characterized in our study, two carried the L213M substitutions in the chromosomal *pmrB* gene, although, according to Lomonaco et al. [37], these mutations seem not to be related to colistin resistance. However, these two isolates showed phenotypic resistance to colistin.

Finally, our findings regarding the K-antigens were consistent with previously reported results for ST307 isolates carrying the *wzi*173 allele, but not for the ST101 isolates, which carried the *wzi*149 allele instead of the *wzi*137 allele [32].

This study has some limitations. First, our data did not cover the entire Puglia region but only three provinces. Nevertheless, the population of the provinces included in this study represents half of the total population of Puglia. A future option would be to implement epidemiological and molecular surveillance of CR-KP infections in the entire region. Second, WGS analysis was conducted on a limited number of isolates. Unfortunately, we could not characterize either the CR-KP strains that carried more than one carbapenem-resistance gene nor all the identified ST307 and ST101 isolates. Nevertheless, we believe that our data could provide important information about the hospital-based dissemination of two emerging high-risk clones, which have, to date, been rarely described in Italy. These clones have characteristics that might lead to increased fitness, persistence in and adaptation to hospital environments and, consequently, an ability to supplant the most frequently reported CC258 clones. Whole-genome sequencing is a promising surveillance tool that will be useful for early identification and characterization of both existing and emerging AMR clones, especially in hospital settings where antibiotic-resistant pathogens pose a great challenge to clinicians due to limited available treatment options. In order to face the antimicrobial resistance, Italy adopted its first National Action Plan on Antimicrobial Resistance (PNCAR 2017–2020) [44], which represents the tool for implementing actions aiming at reducing the AMR at national, regional and local level. The objectives set by the Plan included the monitoring of the antibiotic use and the implementation of prevention and infection control measures. According to the national plan, Puglia region adopted measures of infection prevention and control, aimed at minimizing the transmission and the spread of CR-KP in hospitalized patients. Among these measures, training courses and audit aimed at improving the awareness of health care professionals on AMR were planned. Moreover, a routine screening of patients admitted to hospitals, standardized procedures for management of colonized patients, and an active surveillance of bloodstream infections were implemented. The results of the present study underline the importance to further implement infection control measures in healthcare settings.

## Conclusions

In conclusion, ST101 and ST307 clones are emerging in Southern Italy since they were found in a significant proportion of cases of sepsis. Whole-genome sequencing of isolates revealed the presence of more than one beta-lactam resistance gene. The analysis of virulence genes showed that these clones seemed to be potentially extremely virulent strains. To avoid rapid spread of CR-KP strains, especially in hospital settings, aggressive infection control measures and continuous monitoring for high-risk and potentially high-risk clones as well as effective antimicrobial stewardship strategies are necessary.

## Data Availability

The datasets used and/or analysed during the current study are available from the corresponding author on reasonable request.

## Abbreviations

CR-KP: Carbapenem-resistant *Klebsiella pneumoniae*
MLST: Multi-locus sequence typing
WGS: Whole Genome Sequencing
CRE: Carbapenem-resistant Enterobacteriaceae
CPE: Carbapenemase-producing Enterobacteriaceae
KPC: *Klebsiella pneumoniae* carbapenemase
NDM: New Delhi metallo-beta-lactamase
VIM: Verona integron-encoded metallo-beta-lactamase
OXA-48: Carbapenem-hydrolysing oxacillinase-48
